# Study of monomelic amyotrophy of the lower limbs in the territory of the Western Balkans: Case series

**DOI:** 10.1097/MD.0000000000035435

**Published:** 2023-09-29

**Authors:** Gordana Djordjevic, Vuk Milosevic, Aleksandar Stojanov

**Affiliations:** a Clinic of Neurology, University Clinical Centre Nis, Nis, Serbia; b Medical Faculty, University of Nis, Nis, Serbia.

**Keywords:** case series, limb weakness, monomelic amyotrophy, motor neuron disease

## Abstract

**Rationale:**

Monomelic amyotrophy is a rare form of motor neuron disease in which the neurogenic atrophy is restricted to 1 limb, mostly the distal part of the arm. The disease most often occurs in Asia, especially in Japan and India, while in European countries, this disease is rarely recognized. Registration and publication of new cases of this disease aims to increase the awareness of clinicians about the existence of this disease in European countries, and with the aim of easier recognition and faster diagnosis of this essentially benign disorder.

**Patient concerns:**

Five patients with signs of atrophy of the muscles of 1 leg were examined at our Institution.

**Diagnoses:**

The criteria for selecting patients were as follows: clinical evidence of wasting and weakness confined to the 1 lower limb; progressive course, or initial progression followed by stationary course; absence of any definite sensory loss or central nervous system involved; no evidence of compression lesion of the spinal cord.

**Interventions:**

The clinical characteristics of our patients were similar to those previously described in the literature. The characteristic clinical features were sporadic occurrence, predominance in males with an initially progressive course for 2 to 5 years followed by a stationary state. There was no family history of neuromuscular disease.

**Outcomes:**

The electromyographic finding was consistent with a chronic neuropathic disorder. Magnetic resonance imaging of the lumbosacral spine excluded intraspinal pathologies and root compression in all cases.

**Lessons subsection:**

Monomelic amyotrophy of the lower limb is a rare disease that should be considered in cases of slow progressive unilateral amyotrophy of a single leg, especially in younger and middle-aged men, not only in Asia but also in the Western Balkans and Europe.

## 1. Introduction

Monomelic amyotrophy (MMA) is a rare form of peripheral motor neuron disease characterized by a slowly progressive unilateral amyotrophy in a single limb followed by a spontaneous halt in disease progression. The disease was first described in 1959 as “juvenile muscular atrophy of unilateral upper extremity” when Hirayama et al from Japan reported on 20 cases with patients with slowly progressive unilateral hypotrophy and paresis restricted to the distal aspect of the upper limb.^[1]^ Following the first official publication of this unusual entity, several hundred cases of patients with similar clinical manifestations were reported, predominantly from India and Japan. Sobue et al published the largest series of patients with this disease, reporting findings in 71 patients in 1978.^[2]^ Different authors have described this syndrome under different names, namely, Hirayama disease, benign focal amyotrophy, wasted leg syndrome, monomelic amyotrophy.^[3–5]^ The term “monomelic amyotrophy” was introduced in 1984 to stress the fact that the atrophy was always restricted to a single limb, either upper or lower. Descriptive terms such as “brachial monomelic amyotrophy” or “monomelic amyotrophy of the lower limb” (MMA LL) may be used to specify the type of limb affected. Some authors prefer the term “benign focal amyotrophy” as this term indicates the benign nature of the disease and does not exclude the fact that it could manifest in any limb or the possibility of bilateral involvement of the upper or lower limbs. MMA manifests itself in the form of painless, progressive, asymmetric weakness and atrophy of the muscles in a single limb, usually the distal segments of an arm, while the lower limbs are rarely affected. The first important clinical report of MMA LL was published in 1981.^[4]^ Since then, many cases have been reported, mainly coming from India. The largest series of MMA outside India was reported by Felice et al in 2003 (8 patients).^[6]^ To our knowledge, cases of MMA LL have not been reported on the territory of the Western Balkans. This paper presents 5 patients who were born in Serbia and are still living in the country. All 5 patients presented with unilateral amyotrophy of the lower limb.

## 2. Methods

During 8 years (from 2011 to 2019), 5 patients with signs of atrophy of the muscles of 1 leg were examined at our Institution. The criteria for selecting patients were as follows: clinical evidence of wasting and weakness confined to the lower limb; progressive course, or initial progression followed by stationary course; absence of any definite sensory loss or central nervous system involved; no evidence of compression lesion of the spinal cord. Exclusion criteria were: existence of chronic and systemic diseases; magnetic resonance imaging (MRI) intraspinal pathologies and root compression; history of previous poliomyelitis or a febrile illness, exposure to toxins, heavy metals; positive family history of neuromuscular disease

The study procedures were conducted with approval from the local clinical research ethics committee. All procedures were conducted by the committee guidelines and regulations, including the Basics of Good Clinical Practice, the Declaration of Helsinki, and the Law on Health Care of the Republic of Serbia. All participants provided written informed consent.

A detailed history was obtained, including other chronic diseases, vaccination, exposure to toxins, trauma, viral infections. There was no family history of neuromuscular disease. General physical and neurological examinations were carried out on all patients. Routine laboratory analyses were performed, including complete blood counts, serum electrolytes, creatine kinase, urea, creatinine, and liver and thyroid function tests. Immunologic blood tests, electrophoresis, and immunoelectrophoresis were performed in all patients. One patient underwent cerebrospinal fluid analysis (cytochemical examination and cerebrospinal fluid immunoelectrophoresis). All patients underwent virological analyzes and borrelia burgdorferi. MRI of the lumbosacral spine and electromyoneurography (EMG) was performed. Based on these results a diagnosis of benign MMA LL was made.

## 3. Results

Of a total of 5 patients, 4 were male. The mean age of onset was 33.43 ± 11 (range, 23–48 years). Three patients had suffered a leg injury requiring prolonged immobilization several years before the onset of the first symptoms. The patient characteristics and history are presented in Table 1. Signs of peripheral motor neuron damage in 1 leg were recorded in all patients.

**Table 1:** Clinical characteristics and medical history of patients with monomelic amyotrophy.

No	Gender	Onset-yr	Progression of illness-yr	Side	Leg injury/physical activity	Patient history	Family history
1.	M	24	3	L	+ (5 yr, b.s.)/ +	Non-significant	Non-significant
2.	M	33	3	L	+ (10 yr, b.s.)/−	Non-significant	Non-significant
3.	M	48	5	R	+ (5 yr, b.s.)/−	Non-significant	Non-significant
4.	M	38	5	R	−/−	Non-significant	Non-significant
5.	F	23	3	L	−/+	Non-significant	Non-significant

bs = before symptoms, F = females, M = male, yr = year.

**Figure 1.**
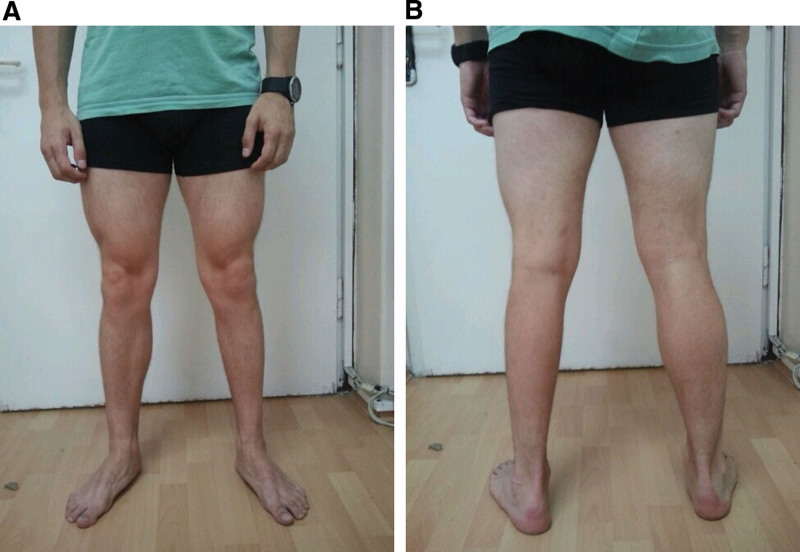
Patient with amyotrophy of the left lower leg: (A) from the front; (B) from the back. (Athletic build of the patient as a result of bodybuilding training).

**Figure 2.**
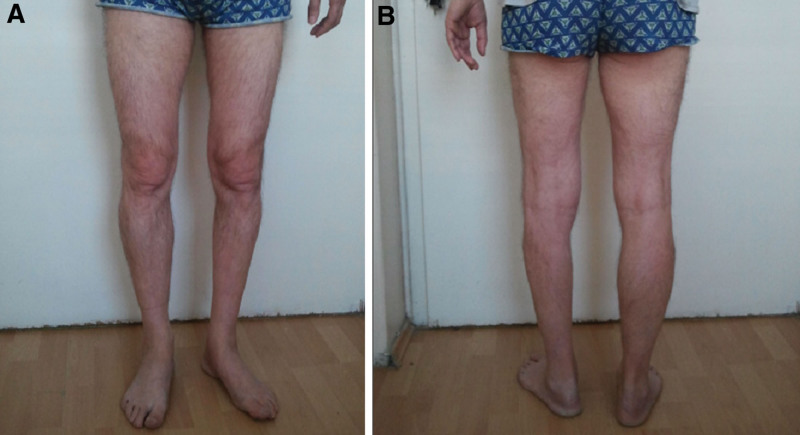
Patient with amyotrophy of the left lower leg: (A) from the front; (B) from the back.

**Figure 3.**
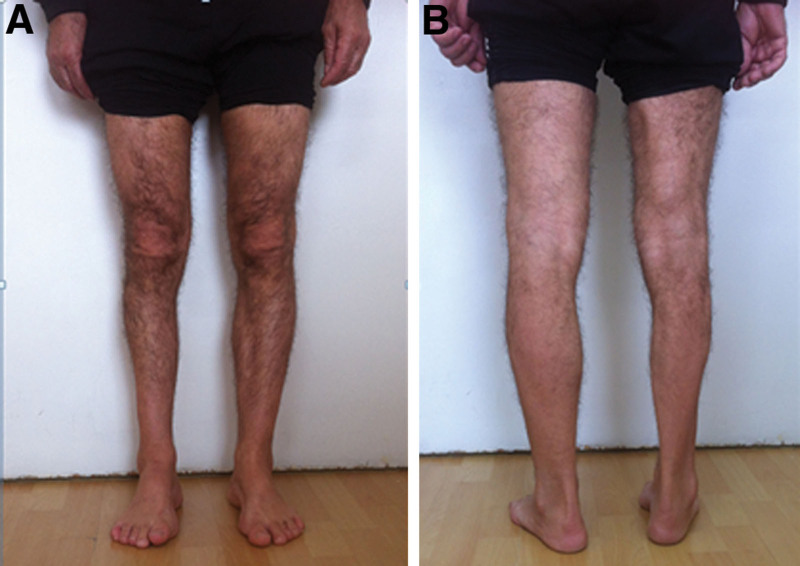
Patient with amyotrophy of the right leg: (A) from the front; (B) from the back.

**Figure 4.**
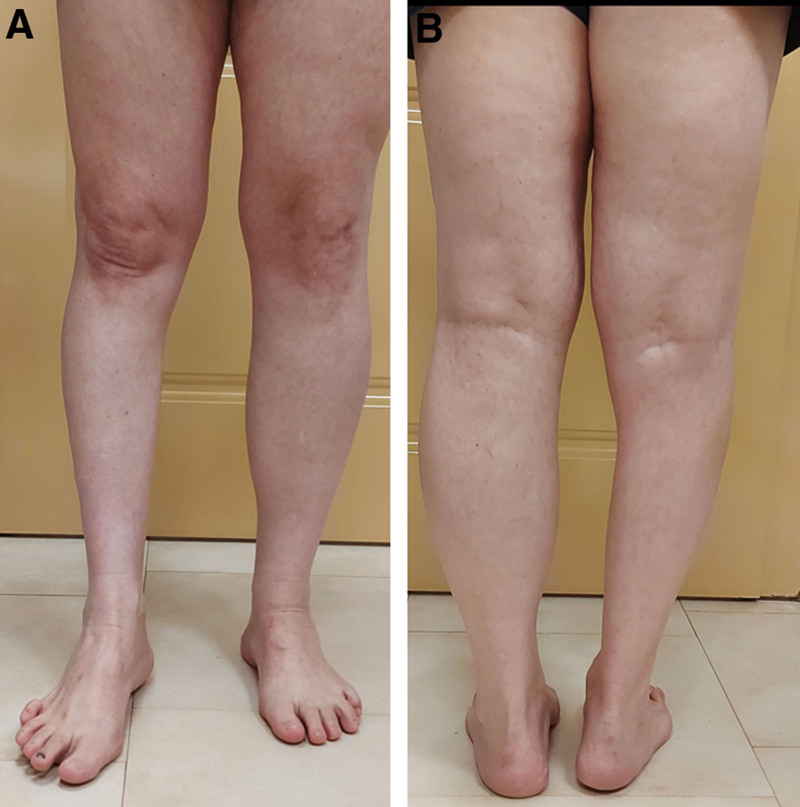
Patient with amyotrophy of the right lower leg: (A) from the front; (B) from the back.

**Figure 5.**
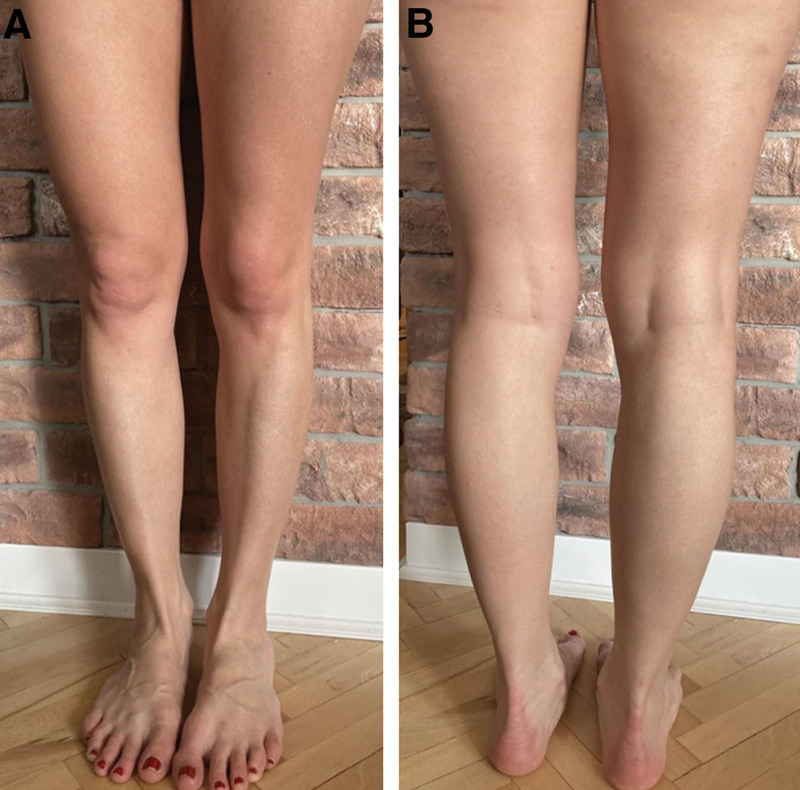
Patient with amyotrophy of the left leg: (A) from the front; (B) from the back.

Three patients were presented with amyotrophy and weakness of the right leg whereas the remaining 2 presented with amyotrophy and weakness of the left leg. One patient presented with unilateral muscle wasting of the lower leg, while other patients presented with hypotrophy of the entire leg. Four of the patients presented with weakness of the distal segments of 1 leg accompanied by difficulty standing on the toes and/or heel of the corresponding leg. The female patient presented with mild weakness of foot dorsiflexion. Neurological findings of our patients are presented in Table 2. Figures 1–5 show clinical manifestations of our subjects.

**Table 2:** Neurological findings of patients with monomelic amyotrophy.

No	Amyotrophy	Muscle weakness	Visible fasciculations	Pes cavus	Tendon reflex P/A	Sensibility
1.	Lower leg	+	+	−	+/−	N
2.	Lower leg and thigh	+	+	−	+ (reduced)/−	N
3.	Lower leg and thigh	+	−	+	+(reduced)/−	N
4.	Lower leg	+	−	+	+/−	N
5.	Lower leg and thigh	+	−	−	+/−	N

A *=* achill reflex, N = normal, P = patellar reflex.

Routine laboratory tests, as well as immunological tests, were within the reference range in all patients without results that would indicate a systemic connective tissue disease, nor dysproteinemia. No patient had diabetes, glucose intolerance or insulin resistance. One patient underwent cerebrospinal fluid analysis and the findings were normal. All patients underwent virological testing and no IgG or IgM antibodies were detected. EMG study results are reported in Table 3. Motor nerve conduction studies in the affected limbs were normal in 4 patients. A decrease in compound muscle action potential amplitude was noted in 1 patient, with normal distal latency and normal conduction velocity. Motor nerve conduction studies in clinically unaffected limbs were normal in all patients. Sensory nerve conduction studies were normal in all patients. Needle EMG studies in the affected muscles showed reduced interference patterns with large motor unit potentials in all patients, as well as evidence of denervation in 3 patients.

**Table 3:** Electrophysiological finding.

No	EMG (MUP)					NCS		
	Denervation (p.s.w., fib./ fasc.)	Recruitment	A	Duration	Polyphasia	DL	NCV	A
1.	+/+	Reduced	Large	Prolonged	+	N	N	N
2.	+/+	Reduced	Large	Prolonged	+	N	N	N
3.	+	Reduced	Large	Prolonged	−	N	N	reduced
4.	−	Reduced	Large	Prolonged	−	N	N	N
5.	−	Reduced	Large	Prolonged	−	N	N	N

A *=* amplitude, DL = distal latency, EMG = electromyography, fasc = fasciculations, fib. = fibrillation potentials, MUP = motor unit potentials, N = normal, NCS = nerve conduction study, NCV = nerve conduction velocity, p.s.w. = positive sharp waves.

An ultrasound examination of the posterior lower leg and upper leg was performed on 1 patient. The results showed that the right gastrocnemius muscle was reduced in volume, had thinned muscle fibers, and was of altered structure and echogenicity, indicating muscle atrophy with the replacement of muscle fibers by fat. Slight asymmetry and changes in similar echo characteristics were observed in parts of the biceps femoris muscle as well (posterior lobe of the upper leg laterally). There was no asymmetry or changes in the morphology of the skin and subcutaneous tissue of both legs.

## 4. Discussion

MMA is a rare form of motor neuron disease in which the neurogenic atrophy is restricted to 1 limb, most often the distal part of the arm. There are rare cases where weakness and atrophy occur in the lower limb or only in the calf muscle.^[7–9]^ The disease is most prevalent in Asia, especially in Japan and India, though it has been less well recognized in European and North American countries. Only in the last 2 decades have individual cases and smaller series of cases been reported in these regions. The most extensive series of MMA outside India was reported by Felice et al in 2003 (8 patients).^[6]^

In this paper, 5 patients with lower limb amyotrophy are presented, 4 men and 1 woman, which is by the male predominance found in the literature. Namely, the disease affects men almost exclusively.^[1,2,10,11]^ In a study by Yoo Yong Kim et al, MMA patients with lower limb involvement were mostly male (8 men and 3 women), unlike the study of De Freitas et al, where there was no male preponderance (8 males and 8 females).^[9,12]^

The disease primarily affects younger individuals. In most patients, symptoms develop in the second and third decades of life. The mean age of onset in MMA patients with upper limb involvement is somewhat lower (19.8) when compared to 28.5 years in patients with lower limb involvement.^[10]^ The mean age of onset of our patients was slightly higher and it was 34.6 years (range 23–50 years), which is by the results reported by Gourie where the mean age was 33.43 ± 11.53, 53 years. In a study by Felice et al, the mean age of onset in North American patients was higher and was 53.3 years (range, 36–84 years).^[6]^

The disease is characterized by a slowly progressive course with a progression of symptoms within 5 years, followed by a spontaneous halt in progression and a stabilization of symptoms, however, without improvement. On average, the progression of the disease in our patients was 3.5 years with a spontaneous halt in progression, and the condition remained unchanged during further clinical follow-up (3–8 years).^[11]^

Leg involvement may be diffuse or limited to a specific group muscle such as M gastrocnemius or M tibialis anterior.^[13–15]^ In 2000, De Freitas et al published a study with 21 patients with benign monomelic amyotrophy.^[16]^ Of 16 patients with lower limb involvement, diffuse atrophy of the leg was observed in 6 patients, amyotrophy was distal in 9 and proximal in 1 case. In our study, 4 patients had diffuse leg atrophy, while 1 patient had lower leg atrophy.

Despite these atrophies, muscle strength is relatively well preserved.^[13,14,16]^ In a study by Gourie et al with 44 patients, 68.2% presented with mild weakness. In our study, all patients presented weakness of the distal segments of the affected foot, with impaired dorsiflexion of the foot, while 1 patient had impaired plantar flexion of the said foot accompanied by the inability to stand on the heel and toes of the affected foot. In addition to the atrophy, patients may experience other symptoms and signs such as fasciculations, abnormal sympathetic skin response, hyperhidrosis, and deterioration in muscle weakness when exposed to cold.^[15,17]^

Muscle reflexes may be normal or absent^[9]^ In our study, 4 patients had an extinguished Achilles reflex of the affected leg, while the female patient had a present but diminished Achilles reflex when compared to the healthy side. The patellar reflex was present in all patients but was diminished in 2 patients with amyotrophy of the entire leg.

Even though the disease is unilateral in most cases, electromyographic examinations often display a neurogenic curve in the muscles of the opposite limb.^[18–23]^ In addition to the clinical findings, EMG plays a key role in diagnosing the disease. EMG examination shows chronic neurogenic changes in the affected muscles, with or without evidence of active denervation (fibrillation, positive sharp waves, fasciculations). These changes may be observed not only in the affected muscles but also in the clinically unaffected segments of the same limb or contralateral limb.^[9,16]^ Electroneurography usually reveals nerve conduction within the normal range; however, it may detect decreased amplitudes of the muscle action potential, slowed motor nerve conduction velocities, and prolonged F-wave response latencies.^[13]^ Electromyographic examination revealed a chronic neurogenic curve in the affected muscles in all 5 of our patients, characterized by a reduced innervation pattern with potentials of higher amplitude and prolonged duration accompanied by a higher percentage of polyphasic potentials in individual insertions. Three patients presented with signs of active denervation process in the muscles of the affected leg in the form of positive sharp waves and fibrillation. Fasciculation potentials were also recorded in 2 of the 3 above-mentioned patients. A neurogenic curve was also observed in the muscles of the opposite limb but of a mild degree of involvement when compared to the affected limb, which is in line with the results presented in the literature.^[18–21]^ As for denervation potential, the patients did not present with signs of active denervation process in the muscles of the opposite leg, unlike the results of a study conducted by Gourie-Devi et al.^[5]^ In their 10 cases of lower limb atrophy, 2 patients showed denervation in the contralateral limb. Some authors have made similar observations.^[20,22,23]^

When undertaking examination on patients with unilateral atrophy and weakness of 1 limb, it is necessary to perform MRI or computer tomography imaging of the spinal cord to exclude other pathological conditions that may manifest similar symptoms, such as spondylosis, disc herniation, syringomyelia, tumors, AV malformations, etc. In patients with MMA of lower limbs, computer tomography and MRI of the lumbar spine and nerve roots do not reveal signs of pathological changes, unlike patients with MMA with upper limb involvement who present with changes in the form of corrected cervical lordosis, focal atrophy of the lower cervical segments (localized lower cervical cord atrophy, asymmetric cord flattening, and loss of dural attachment).^[23–27]^ In our patients, MRI of the lumbar spine spine revealed no significant changes, apart from moderate degenerative changes in terms of compression of nerve structures.

Pathohistological examinations of the affected muscles indicate typical neurogenic atrophy with atrophic and hypertrophic fibers, with secondary myopathic characteristics.^[5,19,20,28]^ Our patients were not encouraged to undertake the pathohistological examination. An ultrasound examination of the posterior lower leg and upper leg was performed on 1 patient. The findings indicated muscle atrophy with the replacement of muscle tissue with adipose tissue.

There are no official reports of postmortem examinations on patients with MMA LL in the literature. However, the results of postmortem examinations on patients with MMA of the upper limbs reveal atrophic changes, necrosis, loss of anterior spinal cord horn cells, and moderate gliosis in the cervical segments. The etiopathogenetic mechanisms that lead to the development of this disease are still unknown. Various factors that can contribute to the development of the diseases such as viral infection, atopy, vascular insufficiency of the spinal cord, heavy physical activity, atrophy of the spinal cord as a result of stretching of the spinal cord during flexion of the neck, were considered.^[29,30]^ It is believed that the most important pathogenetic factor for the development of MMA of the upper limbs is the compression of the spinal cord by tight dura.^[27,31]^ Regarding MMA LL, pathogenetic mechanisms have not been officially proposed. However, the fact that the lumbar part of the spinal cord is extremely sensitive to systemic circulatory disorders suggests the possible pathogenetic role of vascular insufficiency of this part of the spinal cord.^[12]^ A possible risk factor reported in the literature is limb injury due to trauma, as well as more strenuous physical or sports activity.^[19,32,33]^ In our study, 3 patients had suffered a leg injury requiring prolonged immobilization 5 to 10 years before the onset of symptoms in the same leg (5 years on average). One of the patients has been doing bodybuilding in recent years. Moreover, the female patient was involved in intensive physical training. Gourie published a study in 1993 that analyzed the risk factors for the emergence of MMA.^[16]^ The data clearly show that heavy physical activity was the most significant factor present with a greater frequency in cases as compared to controls. However, when examined separately, injuries and type of occupation did not show a significant difference between the 2 groups.

MMA LL is a rare condition characterized by a slowly progressive, unilateral amyotrophy in a single limb, usually occurring in younger and middle-aged males. To make a definitive diagnosis, it is necessary to exclude other possible causes of amyotrophy of the lower limb and carefully monitor the course of the disease in further progression to confirm a halt in progression and a stabilization of symptoms. The patients outlined in this paper are presented with characteristic clinical features that correspond to the descriptions found in the literature. What is particularly interesting is that 4 out of 5 patients had suffered severe limb injuries and/or had undertaken strenuous physical exertion several years before the onset of the first symptoms. The potential limitations of this study should be pointed out, which is primarily the short clinical follow-up of patients after the appearance of the first symptoms (3–5 years).

The increase in new case reports from the Western Balkans indicates that this disease is more common than previously thought in these regions. Therefore, when performing diagnostic testing, it is necessary to consider the possibility of this disease in patients with the described clinical symptoms in other European countries as well.

## Acknowledgments

We want to thank to all patients who were enrolled in this study. This paper is drafted within the internal scientific project of the Faculty of Medicine University of Nis, number 451-03-68/2022-14. The work should be attributed to Clinic of neurology Nis.

## Author contributions

**Conceptualization:** Gordana Djordjevic, Aleksandar Stojanov.

**Formal analysis:** Gordana Djordjevic.

**Investigation:** Gordana Djordjevic, Aleksandar Stojanov.

**Writing – original draft:** Gordana Djordjevic.

**Writing – review & editing:** Vuk Milosevic.
